# Chemotherapy only in early-stage Hodgkin lymphoma: More relapses but “same” (or possibly worse) survival – Reconsidering the misguided trend to omit radiotherapy

**DOI:** 10.1007/s11899-014-0222-5

**Published:** 2014-06-18

**Authors:** Joachim Yahalom

**Affiliations:** Memorial Sloan-Kettering Cancer Center, 1275 York Avenue, New York, NY 10065 USA

**Keywords:** Hodgkin Lymphoma, Chemotherapy alone, Combined modality therapy, Radiotherapy, Involved-site radiotherapy (ISRT)

## Abstract

The standard treatment of early-stage Hodgkin lymphoma (ESHL) as recommended by most national guidelines is combined modality treatment (CMT) that includes a short course ABVD followed by a small field of low dose radiotherapy (RT). Recently a trend to treat patients with more chemotherapy alone has been promoted by some claiming that chemotherapy alone is good enough, and the overall survival is similar. These arguments need to be carefully examined, and the risk of more chemotherapy upfront and salvage considered. The suggestion that interim PET will identify patients that can have similar results with chemotherapy alone has recently been questioned by the results of both European and UK studies. It is the subject of this critical review.

## Introduction

Treating highly curable early-stage Hodgkin lymphoma (ESHL) patients with *chemotherapy alone* has recently gathered more steam in North America. Yet, omission of radiotherapy (RT) is not considered a standard treatment for ESHL in Europe [1••] or in the UK [2••] Since it is almost guaranteed to result in worse disease control compared to combined modality therapy [3]. Notwithstanding, US national statistics indicate that an increasing number of patients with early HL are not receiving combined modality therapy [4••]. The recognition that combined modality is the tried and true standard treatment is clearly spelled out in recent updates of several national guidelines (NCCN, UK, and ESMO). Some have caveats concerning treatment with chemotherapy alone or consider it still experimental [1••, 2••, 5]. The increasingly popular trend to treat HL with chemotherapy alone is worrisome for several reasons, including the possibility that we may see, for the first time in decades, as the data indirectly suggest, a reversal of the improvement in outcome of ESHL patients.

In this article, I will critically analyze data from recent prospective randomized studies and refute arguments made in support of omitting RT, often while substituting it with more chemotherapy.

## US data indicate a decrease in the use of RT for ESHL and worse OS for patients treated with chemotherapy alone

Recent studies analyzed information on ESHL patients from two large US databases. Koshi et al., analyzed the Surveillance, Epidemiology, and End Results (SEER) database [4••]. They identified 12,247 ESHL patients diagnosed between 1988-2006 (median follow-up 4.9 years) and showed that while during the years 1988-1991 63 % of patients received RT as part of their initial treatment, in 2004-2006 only 44 % received RT, (*p* < 0.001). The 5-year OS for ESHL that *did not* receive RT was 76 %, significantly inferior when compared to those who have received RT obtaining a 5-year OS of 87 % (*p* < 0.001). The hazard ratio adjusted for other variables in the regression model showed that patients who did not receive RT (HR – 1.72, 95 % CI – 1.72–2.02) was associated with significantly worse survival when compared to patients who received RT. Interestingly, the study showed that the actuarial rate of developing a second malignancy at 15 years was 14.6 % with RT vs 15.0 % without RT. Similarly, a recent, yet unpublished study by Parikh et al. (to be reported in ASTRO September 2014), the National Cancer Data Base (NCDB) was used to identify over 40,000 patients with ESHL from more than 1,500 sites across the US. It has shown similar results to the SEER data. Namely, a decrease in the use of RT in ESHL over the last decade, and unfortunately, a highly statistically significant inferior overall survival for the group treated with chemotherapy alone compared to those treated with combined modality. This effect on OS was independent of other prognostic factors in a multivariate analysis.

## The tangled relationship between freedom from treatment failure and overall survival in HL

Historically, even the most important randomized studies that established principles of treating HL in different eras were judged primarily on the capacity of the chosen arm to obtain a significantly better freedom from treatment failure without undue toxicity. In almost all studies, due to salvage options or a small number of mortality events, OS remained without a demonstrated advantage to the chosen, better disease controlling arm. A few of many examples in ESHL are studies of extended-field RT (EFRT) alone versus involved-field RT (IFRT) alone [6], STLI vs. AV + STLI (SWOG 9133/CALGB 9391) [7], STLI vs. ABVDx2 + STLI (GHSG HD7) [8], and EBVPX6 + IFRT vs STLI (EORTC H7F) [9]. There are many more; and, indeed, showing OS advantage in HL randomized studies is a rarity.

There are several reasons for reporting the “same” OS in spite of better freedom from treatment failure:

1. *Effective salvage*
  Patients who have failed their primary treatment may be salvaged with high dose chemoradiotherapy and stem cell transplantation. The potential devastating effects of this intervention will be discussed later.
2. *Long survival with active disease*
  Many patients can survive with active disease for several years with temporizing treatments like single agent vinblastine or palliative RT. A death event may take many years to occur.
3. *Death from other causes*.
  Overall survival captures death from any cause, and the more appropriate parameter to look at should be cause specific survival (CSS) that includes death from disease, death from complications of salvage, and death from complications related to treatment such as death from leukemia, other lymphomas or second solid tumors in the radiation field (related often to chemotherapy, radiotherapy or both), heart failure and /or coronary heart disease (chemotherapy and/or RT). Obviously, deaths from Alzheimer’s disease, suicide or drowning as counted on the combined modality-arm in HD 6 may be misleading (see below) [10••].

To reach a statistical relevance in a noninferiority study and avoid a type II statistical error in analyzing OS or even CSS, the study should have a sufficient number of mortality events. Fortunately, this is quite rare in early-stage HL and practically all studies in the modern era have not had adequate death events to reach a statistical difference. Furthermore, most studies have been reported after a median of 3-7 years and collaborative group studies typically lack funding to maintain an accurate long-term follow-up and determine cause of death even when they are revisited later.

In order to bypass some of the obstacles of limited statistical power of individual randomized trials, the Cochrane group analyzed all randomized controlled trials (RCT) comparing chemotherapy alone to combined modality therapy in early-stage HL.[3] Five RCTs involving 1,245 patients were included. The HR was 0.41 (95 % confidence interval (CI) 0.25 to 0.66) for tumor control and 0.40 (95 % CI 0.27 to 0.61) for OS for patients receiving CMT compared to chemotherapy alone. Complete response rates were similar between treatment groups. In sensitivity analyses another six trials were included that did not fulfill the strict inclusion criteria of the Cochrane group protocol but were considered relevant to the topic. These trials underlined the results of the main analysis. The Cochrane group concluded that adding radiotherapy to chemotherapy improves tumor control and overall survival in patients with early-stage Hodgkin lymphoma.

## The puzzling results of the Canadian HD6 trial

In synopsis, this is a trial that randomized RT alone, ABVD alone, and ABVD followed by extended (and outdated) field RT in favorable and less favorable (but without bulk or with B symptoms) early-stage HL patients [10••]. The study showed a significantly better disease control using combined modality using two cycles of ABVD followed by the long abandoned subtotal lymphoid (spleen included) RT field (STLI) compared to the group that received double and often triple the ABVD dose alone (HR = 3.2; *p* = 0.006). Yet, surprisingly, more mortality events (23 patients) occurred in the less favorable group that received ABVD x2 + STLI than in the ABVD alone group (11 patients) resulting in a better overall survival (*p* = 0.04). Although this radical RT was abandoned more than two decades ago, several opinion leaders argued that RT should be eliminated regardless of the advantageous disease control and in spite of the fact that modern RT uses a very small field that includes only the involved site and lower doses. Interestingly, the favorable patients that received STLI alone in HD6 had no reported long-term mortality and minimal morbidity.

HD6 also serves as an excellent example how a small number of events, unrelated causes of death, and incomplete analysis of morbidity may distort the results, conclusion, and interpretation (Yahalom J: *Favorable Early*-*stage Hodgkin Lymphoma and HD.6*: *The take and the don*’*t take home messages*. ASCO POST January 15, 2012, Volume 3, Issue 2 and Engert A; *Radiotherapy in early*-*stage Hodgkin lymphoma*, ASCO POST June 15, 2012, Volume 3, Issue 9). Briefly, one of the caveats is that all mortality events (5) titled “others” have peculiarly occurred only in the ABVD/STLI cohort. They included Alzheimer’s disease, accidental drowning, and suicide. In addition, three deaths “related to infection” were again noted only in the ABVD/STLI arm. Perhaps these deaths were related to unnecessary irradiation of the spleen in STLI; but in practice, it is extremely rare to see lethal infections related to radiation of HL. Remove even only the “other” deaths from the analysis and the OS in HD 6 is with no difference in survival between the arms, showing only better tumor control with the combined modality arm. Other issues of concern for HD6 are detailed in the references cited above.

## The Unfulfilled Promise of Interim PDG-PET Scanning

With the hope that negative FDG-PET after two cycles or three cycles of ABVD will identify a group of patients that will achieve only minimally inferior freedom from treatment failure (FFTF) with chemotherapy alone, prospective randomized noninferiority studies were designed. A consortium of three collaborative groups EORTC, GELA, and IIL designed the H10 favorable (F) and unfavorable (U) studies [11] in the UK. The NCRI lymphoma group designed the RAPID study [12••].

The H10F and H10U were designed as noninferiority studies that allowed up to 10 % difference in FFTF between the randomized arms to be considered acceptable, namely showing that omitting RT is acceptable. Only patients that achieved a negative PET status after two cycles of ABVD could be randomized to either an additional cycle of ABVD followed by involved site RT (ISRT) - the “standard” treatment - for the favorable patients (H10F), or to the experimental treatment that replaced ISRT with additional cycle of ABVD (total of four). For the unfavorable ESHL patients who were PET(2)-negative, the “standard” arm consisted of a total of four cycles of ABVD followed by ISRT. The experimental arm replaced ISRT with two additional cycles of ABVD (total of six).

Surprisingly, due to an unexpected number of events (i.e., relapses) occurring before the H10 studies finished their planned accrual; an interim analysis was mandated by the Internal Data Monitoring Committee of the study. At that time, 1,137 were studied and 34 events occurred in the negative PET arms. The investigators decided to terminate the no-RT arms and mandated that all patients since the interim data analysis will receive only standard arm that included RT [11]. The reason was an excessive number of failures on the no-RT arms of both the H10F and H10U. Testing for futility in this noninferiority trial showed that noninferiority of the arms (even if allowing for 10 % difference for negative PET patients) could not be achieved. After two cycles of ABVD, 85.8 % in H10F had a negative PET. On the combined modality of H10F, there was only one event, but nine events with chemotherapy alone. On H10U 74.8 % had an early negative PET with seven events on the combined modality arm and 16 events with more chemotherapy alone [11].

The UK RAPID trial included ESHL patients without B-symptoms or mediastinal disease. There were 420 patients who were negative after three cycles of ABVD. They were randomized to receive 30 Gy of involved field RT or no further treatment [12••]. RAPID was also designed as a noninferiority trial and the bar was set at 7 % difference between the arms. The results are conflicting and depend on the parameters for analysis. If patients are analyzed according to the initial randomization, but not according to the real treatment received, there was no statistically significant difference between the arms. But since a substantial number of patients (26 of 211) on the RT arm, have never received RT, including five patients who died and one who developed pneumonia shortly after ABVD have not received any RT, but were scored as RT arm events. Appropriately, an “as treated” analysis was performed (but not reported in the meeting abstract). With this more realistic analysis, the 3-year PFS was 97 % for the combined modality arm compared to only 90.7 % with chemotherapy alone (HR = 2.39, [13], *p* = 0.03). Thus, in this study, like H10F and H10U, omitting RT even for negative PET led to an inferior disease control. Obviously, it is too early for meaningful survival comparisons. Another important caveat in the interpretation of the RAPID study on the relative prediction of a “negative” PET is that, in this strictly monitored effort of the RAPID team, only high quality imaging data were allowed, and those have been analyzed by only two experts at one central review center using a conservative definition of PET negativity [14]. Not necessarily the kind of PET imaging and analysis that is coming to an imaging facility near you.

## Omitting RT equals receiving more chemotherapy, higher need for salvage, and potentially inferior quality of life

When ABVD alone is offered as an alternative to ABVD + RT, it normally means that the number of ABVD cycles and, thus, total dose of each agent will be doubled or tripled [10••]. This was the case in the HD6 trial and in the H10F or H10U studies. At the same time, the German Hodgkin study group in their prospectively randomized trial of HD10 showed that only two cycles of ABVD followed by 20 Gy IFRT in favorable ESHL sufficed to achieve excellent FFTF and OS that was similar to the previous standard of ABVD X4 and IFRT of 30 Gy [15••].

Subjecting 100 % of patients to more ABVD may be risky and unnecessary. ABVD alone (with no RT) was shown to be associated with significantly more deaths from myocardial infarction [13] as well as under reported but clinically significant bleomycin induced lung injury [16]. As suggested by the RAPID study toxic death events after only ABVD, the risk may be particularly high in elderly patients [17].

Mental and psychological long-term complications of chemotherapy have not been well studied, but have been the subject of concern for breast patients who received adjuvant chemotherapy and discussed first in patient chat rooms and survivors groups under the title “chemo head”. Only recently, prospective functional MRI studies documented that changes in brain activity may underlie chemotherapy induced cognitive complaints in early-stage breast cancer patients receiving adjuvant chemotherapy [18••]. This is a new concern for our young and old patients exposed to more courses of chemotherapy as initial treatment and in salvage when RT is omitted. It should be explored further.

Thus, it is likely that 100 % of the patients that will be advised against RT will receive more ABVD than in the “2 by 2” approach following the GHSG HD 10 program of only ABVD X2 and 20 Gy IFRT that is recommended as the standard treatment for favorable patients [15••]. Unfavorable patients will receive of ABVD X4 and IFRT of 30 Gy proved highly effective by the GHSG HD 11 rather than ABVD X6 and had a higher risk of failure [19••].

Chemotherapy alone program will also fail approximately 10 % more favorable patients when compared to a combined modality program, and even a higher fraction of unfavorable patients may fail without RT particularly if with bulky disease.

Most of these patients will require salvage with regimens like ifosphamide, carboplatinum, and etoposide (ICE), brentuximab vedotin, larger fields, and higher dose of RT to the refractory/relapsing sites followed by high dose chemotherapy and autologous stem cell transplantation. Although salvage is quite effective, its potential conern is for immediate and long-term toxicity being high. The emotional trauma and mental price for these unfortunate (but mostly avoidable or more) 10 % patients is hard to quantify. Many will lose their fertility and will disrupt their young life plans of studies and family building. Even if there is only 10 % risk, is omitting RT justified?

Adding RT was blamed in the past as a cause of long-term morbidity. Most of the data come from the era when RT was used as a single modality, and, at that time, it was the best curative option if given in maximal dose (over 40 Gy) and covering all lymph nodes sites from the top of the neck to the groin, included the spleen and was called, “radical radiotherapy” or total lymphoid irradiation. Many normal organs were indiscriminately irradiated with this ancient technique, it included the breasts, lungs and heart. This has long been abandoned (with the exception of its use in the Canadian HD6 study). The smaller fields used safely in GHSG HD10 and HD11 and the even smaller involved node RT (INRT) practiced in the EORTC/GELA/IIL H10F and H10U reassure us of their efficacy [20]. The modern guidelines published by the International Lymphoma Radiation Oncology Group (ILROG) detail the new approach of smaller Involved-Site RT (ISRT) that can be simply implemented by radiation oncologists [21••].

To reduce the scare from RT that has flourished over the last couple of decades, one should recognize that when the breast is not exposed to radiation (as is possible today in most patients), the risk of breast cancer is not elevated [22]. Even those young patients treated years ago and who had breast exposure are now detected very early with the use of breast MRI combined with mammography. In a recent prospective imaging trial, almost all breast cancers were detected as in-situ lesions or less than 1 cm in size and are fully curable [23].

The other scare issue was the slightly higher risk of RT-related coronary heart disease. Interestingly, some studies showed that the increased risk is mostly detected in patients with other cardiac risk factors (blood pressure, hyperlipidemia, smoking, and diabetes) and rarely is an issue in a cardiac healthy patient [24]. Today, we proactively monitor other risk factors, prescribe statins and reduce other risk factors. The awareness of this relatively minor risk in follow-up provides an added benefit to HL patients.

## What to do?

ESHL patients today should benefit from a well established effective and safe mini-ABVD and reduced dose mini-field RT using simple but modern RT technology and concepts as established by GHSG HD 10 and 11. I am concerned about the 100 % of patients that are likely to receive more ABVD instead of mini-RT and still be at least at a 10 % risk of failure and need for salvage high dose therapy.

The interim PET studies confirmed that even negative PET patients are more likely to fail without RT, yet this group may be smaller.

The 2014 UK guidelines addressed the exceptional option of chemotherapy alone properly by recommending that if chemotherapy alone is considered, the patient should also have a discussion with a radiation oncologist to hear about the pros and cons of RT in her/his particular case [2••] Unfortunately, these referrals rarely happen in North America and many radiation oncologists meet the patients only at the time of chemotherapy failure. There are individual cases that will benefit from the chemotherapy alone approach and that could be determined by radiation oncologists who can identify the young females who may require RT to a site where breast tissue cannot be spared from RT. This is how a lymphoma team should approach an individually tailored curative treatment in 2014. Generalizations, dogmas, and scare are the ways of the past.
